# The morphology of the femoral footprint of the anterior cruciate ligament changes with aging from a large semicircular shape to a small flat ribbon-like shape

**DOI:** 10.1007/s00167-022-06935-2

**Published:** 2022-03-22

**Authors:** Rodolfo Morales-Avalos, Simone Perelli, Félix Vilchez-Cavazos, Tadeo Castillo-Escobedo, Víctor M. Peña-Martínez, Rodrigo Elizondo-Omaña, Santos Guzmán-López, José Ramón Padilla-Medina, Juan Carlos Monllau

**Affiliations:** 1 Knee and Arthroscopy Unit (ICATKNEE-EQUILAE), Department of Orthopedic Surgery, Institut Catalá de Traumatologia i Medicina de l’Esport (I.C.A.T.M.E.), Hospital Universitari Dexeus, Universitat Autonoma de Barcelona (U.A.B.), Barcelona, Catalunya Spain; 2 Department of Surgery and Morphologic Science, Orthopaedic Surgery Service, Hospital del Mar, Universitat Autònoma de Barcelona, Barcelona, Spain; 3 Knee Unit, Department of Orthopedic Surgery and Traumatology, School of Medicine and University Hospital “Dr. José Eleuterio González”, Universidad Autonoma de Nuevo León (U.A.N.L), Monterrey, Nuevo León México; 4 Department of Human Anatomy, School of Medicine , Universidad Autonoma de Nuevo León (U.A.N.L.), Monterrey, Nuevo León México

**Keywords:** Anterior cruciate ligament, Aging, Area, Attachment, Femoral, Footprint, Insertion site, Morphology, Shape, Variations

## Abstract

**Purpose:**

Compare the differences in the morphology of the ACL femoral footprint between the cadavers of the young and elderly in consideration of the degenerative physiological process that occurs with aging.

**Methods:**

The femoral footprint of the ACL was dissected in 81 knees of known gender and age (45 male/36 female). They were divided into four groups by age and gender, establishing 50 years as the cut-off point to divide patients by age. Three observers analyzed the femoral footprint dissections, and the shapes were described and classified. The area and morphometric characteristics of the femoral insertion of the ACL were determined and these were compared between genders and age groups.

**Results:**

The femoral footprint of the ACL from the cadavers of males younger than 50 years of age presented a semicircular morphology in 90% of the cases. In males aged more than 50 years, a ribbon-like morphology was found in 96% of the cases. In women less than 50 years old, the semicircular morphology was observed in 93.7% of the cases. In women aged over 50 years old, the ribbon-like morphology was found in 95% of the cases. A significant difference was observed between the prevalence rates of the morphologies, area size and measurements of the younger and older groups (*p* < 0.001 for both genders).

**Conclusions:**

The femoral insertion of the ACL presents variations in its morphology, area and morphometric characteristics over time. It goes from a large semicircular shape that almost contacts the posterior articular cartilage to a smaller, flattened ribbon-like shape that moves away from the edge of the articular cartilage. It is bounded anteriorly by the lateral intercondylar ridge. These findings should be considered to avoid employing reconstruction techniques in which femoral tunnels with oval or rectangular shapes are used in patients under 50 years of age because they do not correspond to the morphology of the femoral insertion of the ACL in this age group.

## Introduction

Anatomic anterior cruciate ligament (ACL) reconstruction attempts to recreate the native dimensions of the ACL, collagen orientation and insertion sites to recreate the function of the native ligament [32, 42]. It has already been shown that anatomic ACL reconstruction, in which the graft is placed within the native ACL insertions, might result in better knee stability when compared to nonanatomic techniques [6, 24]. Furthermore, the correct placement of the femoral tunnel is known as one of the most important factors influencing knee kinematics and clinical results in anatomic ACL reconstruction [23, 34].

ACL reconstruction was conventionally performed with a single-round femoral tunnel. However, some anatomical studies have revealed that the ACL femoral footprint is relatively elliptical in shape, not round [19]. Therefore, the use of bi-fascicular techniques or the realization of rectangular or oval tunnels have been proposed to try to imitate the native anatomy of the femoral insertion of the ACL in a more precise way. Still, some studies have described morphologies of the femoral insertion as being crescent-shaped, circular/round [12], semi-circular [10] or ovalized [10, 11]. Moreover, differences in the size of the femoral insertion have been described. A few studies have reported a long femoral insertion that extends backward to the articular cartilage margin [3, 7, 12, 18, 29], whereas other studies have described a relatively narrow insertion area [11, 26, 40].

Due to this confusion, a large number of anatomical studies have been carried out that have tried to precisely define the morphological characteristics of the femoral insertion of the ACL [7, 10–12, 16, 19, 34, 39, 40]. However, most of those studies were carried out with small samples and with elderly cadavers, which could lead to errors in the determination of the true morphological characteristics of the ACL. In addition, on other occasions, they do not make a clear distinction between the analysis of the direct insertion (mid-substance insertion) and the indirect insertion (fan-like extension) of the ACL.

A recent study determined that the tibial insertion of the ACL presents significant morphological variations with age [27]. Therefore, it is thought that similar changes may occur at the femoral insertion. The aim of the study was to compare the differences in the morphology of the ACL femoral footprint between the cadavers of the young and elderly due to a degenerative physiological process that occurs over time. The hypothesis is that there are significant variations in the shape of the femoral insertion of the ACL between the cadavers of younger people and older people. The results of this study will help to determine the normal shape and position that the femoral tunnel should have in a more individualized way.

## Materials and methods

This study was approved by the Institutional Review Board and Ethics Committee of the School of Medicine and University Hospital “Dr. Jose Eleuterio Gonzalez” of the Universidad Autonoma de Nuevo Leon with registration number OR16-00007. The study was carried out in accordance with the World Medical Association Declaration of Helsinki of 1964 and its revision in 2013 [45]. An anatomical, descriptive, observational and comparative study was performed with 88 non-paired, formalin-fixed human cadaver knees from the Mexican population is included.

The cadavers were collected from three distinct departments of human anatomy between 2016 and 2020. All specimens were over 18 years of age at the time of death. The gender, weight, and height of each cadaver were documented. The cause of death and the most important clinical information for each corpse was known and varied between the cadavers of the young and elderly. Head trauma was predominant in the young group and myocardial infarction and metabolic disorders in the elderly group. Unequivocally so, none of the causes of death or medical history of the included cadavers influenced the execution and results of this study.

The sample size was determined based on the availability of cadavers of individuals under 50 years of age. Data from a previous study of ACL morphology confirmed that the sample size was sufficient to detect differences among ages [27]. Furthermore, a post-hoc power analysis was performed to assess the adequacy of the sample size. Additionally, the goal of the study was for it to be done with the largest sample reported in the literature. The sample was divided into four groups according to age and gender: males and females younger and older than 50 years. This cut-off age was used based on a previous study that showed statistically significant differences in the tibial footprint morphology of the ACL [27].

All the knees were analyzed in phases to make sure they fulfilled the inclusion criteria. Before being included in the study, every specimen underwent an anteroposterior radiograph to exclude knees that presented an intraarticular fracture sequela, the presence of orthopedic implants or congenital bone anomalies. However, they were not used for the radiographic classification of osteoarthritis. In the inspection, those that presented scars from previous surgical interventions were excluded. Those knees that, at the time of the dissection, presented an ACL or macroscopic rupture of the posterior cruciate ligament (PCL), advanced osteoarthrosis (Outerbridge Grade IV) or macroscopic degenerative changes in the synovial membrane that impaired adequate observation or dissection of structures of interest were excluded. Furthermore, the presence of osteophytes in the medial region of the lateral femoral condyle, which could interfere with the measurements of distances between the ACL attachment and the articular cartilage of the condyle, was considered criteria for exclusion. A knee surgeon (SP) and an anatomist (SGL) independently reviewed the specimens for inclusion.

## Dissection technique

Prior to dissection, the specimens were submitted to a 10% formaldehyde fixative solution and stored in a mixture of 54% ethylene glycol, 40% ethanol and 6% formaldehyde with colorant (CTR Scientific^®^, Monterrey, Mexico). Subsequently, the specimens were kept for 60 days in liquid glycerin [2].

The dissection technique was standardized and performed in the same way by all the team members to ensure consistency, and was based on our own experience and the dissection protocols reported in previous studies [3, 16, 19, 26, 34, 37, 39, 40]. The team consisted of a knee surgeon (FVC), an anatomist (REEO), and a senior orthopedic surgery and traumatology resident (JRPM).

The limbs were amputated at two sites, the first at the level of the thigh at 30 cm from the knee joint line and the other at the leg at 20 cm from the knee joint line. During the dissections, the knees were placed in a mechanical press that kept the knees in full extension and made flexion at 30°, 60° and 90° possible without any translational or rotational forces.

All superficial soft tissues on the anterior, posterior, medial and lateral surfaces of the knee were removed up to the joint capsular plane. The entire extensor apparatus was resected distally from the anterior tibial tuberosity and subsequently removed. After exposing the anterior side of the knee joint, the synovial tissue and the Hoffa fat pad were carefully dissected and separated from the joint soft tissue structures (meniscus and transverse ligament). The entire joint capsule, tibial and fibular collateral ligaments, menisco-femoral ligaments, posterior cruciate ligament and synovial tissues were carefully excised.

Starting from the uppermost point of the intercondylar notch, the femur was separated along the sagittal plane with an oscillating saw (Precision 7, Stryker^®^, Madrid, Spain). This was done to observe the full medial wall of the lateral femoral condyle and the lateral intercondylar ridge (resident’s ridge). Care was taken to avoid injury to the ACL or its bony insertion. Exposure after the medial condylectomy made for an excellent view of the femoral attachment of the ACL. The anteromedial and posterolateral fascicles (AM and PL) were identified by observing the orientation of the fibers and the differences in the tension pattern in the range-of-motion. The AM bundle fibers become taut during flexion, while PL bundle fibers become taut by extension. It is important to mention that it was not possible to show a division into fascicles in all cases. Thus, it was only done on specimens where it was possible. It is worth noting that it was particularly difficult in the elderly specimens. The synovial envelope of the ACL bundles was carefully excised with a surgical blade and then sutures with different colors were passed around each bundle to identify them when it was possible. A meticulous dissection of the ACL fibers was performed up to their osseous insertion in the lateral femoral condyle. The insertional fibers were carefully separated from the surrounding synovial tissue while registering the presence of the indirect ACL insertion fibers or “fan like” extension fibers by identifying a macroscopically visible fold between the direct and indirect insertion of the ACL [39]. At the same time, the relationship of the femoral insertion to the lateral intercondylar ridge was analyzed using a microsurgical microscope with 10 × magnification (Carl Zeiss OPMI^®^ Pico, Berlin, Germany). To properly identify the footprint without damaging it during dissection, an ink stain was applied on the bone immediately around the periphery of the entire femoral insertion site (direct + indirect portion) by one of the authors who did not participate in the dissections or in the morphological classification of the femoral footprint (VMPM) [16]. Then, the ACL was delicately excised as closely as possible to the intercondylar wall of the lateral condyle in 90° of flexion using a No. 11 scalpel blade. After that step, the previous ink mark was removed. The remnant of the femoral insertion after its transection as well as the previous division of its fascicles served for its later classification.

**Fig. 1:**
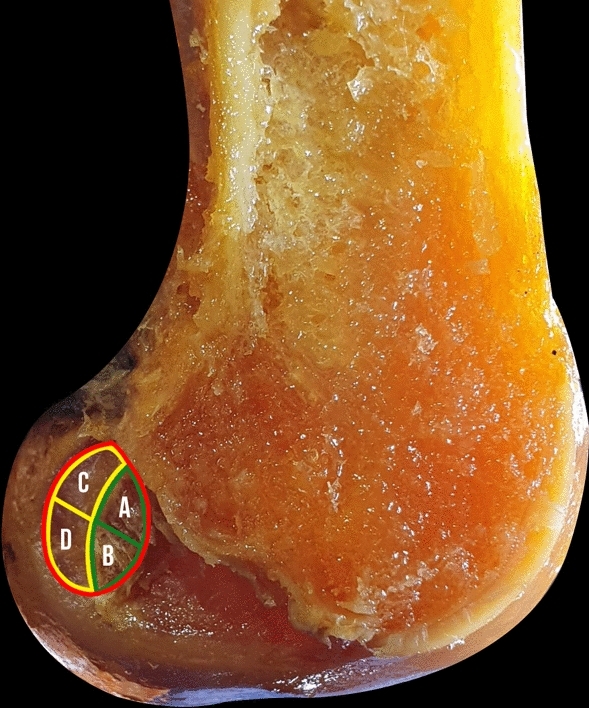
Different areas and zones of the femoral insertion of the ACL in a cadaveric knee from the young group. The whole femoral ACL footprint (red), direct insertion (green), indirect insertion (yellow), the direct insertion area of AM (**A**) and PL bundles (**B**), fan-like extension fibers area of AM (**C**) and PL bundles (**D**)

Following a method previously described, all the different ACL footprint portions were marked with ink: (1) the whole femoral ACL footprint, (2) the direct insertion, (3) the indirect insertion (fan-like extension), (4) the whole AM and PL area, (5) the direct insertion area of AM and PL bundles and (6) the fan-like extension fibers area of AM and PL bundles (Fig. 1) [16]. The points 4–5–6 were marked only when the AM and PL bundles were clearly identifiable.

**Fig. 2:**
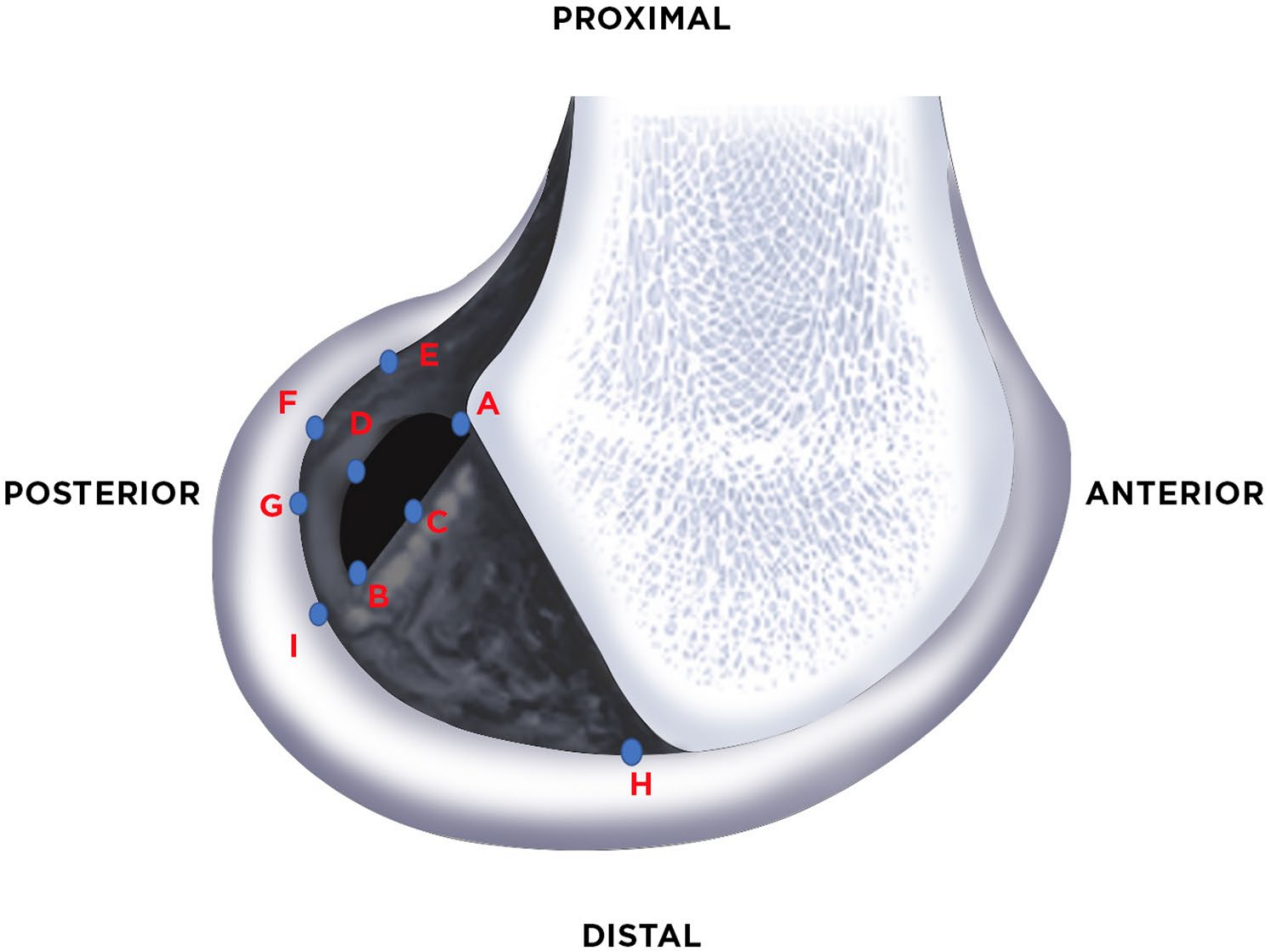
Morphometries performed on each knee like this with the millimeter digital vernier and then were performed a second time using the image J computer program. Proximal border of the direct ACL attachment (**A**), Distal border of the direct ACL attachment (**B**), Anterior extent of the direct ACL attachment (**C**), Posterior extent of the direct ACL attachment (**D**), Border of the posterior articular cartilage in a horizontal line with the proximal border of the direct ACL attachment (**E**), Border of posterior articular cartilage in a horizontal line with the posterior extent of articular cartilage (**F**), Border of posterior articular cartilage in a horizontal line with the distal border of the direct ACL attachment (**G**), Border of the anterior articular cartilage in a horizontal line with the anterior extent of the direct ACL attachment (**H**), Border of the inferior articular cartilage in a vertical line with the distal border of the direct ACL attachment (**I**)

Subsequently, the team of surgeons proceeded to measure the following morphometric parameters using a millimeter digital caliper with a precision of 0.01 mm (Mitutoyo Digimatic w/Absolute Encoders-Series 500, Tokyo, Japan). Figure 2 details each of the landmarks used. The landmarks were defined by consensus in the dissection team when it was done.

1. Proximodistal diameter of the direct ACL attachment area (long axis) (A–B)
2. Anteroposterior diameter of the direct ACL attachment area (short axis) (C–D)
3. Horizontal distance between the proximal border of the direct ACL attachment and the adjacent margin of the articular cartilage (A–E)
4. Horizontal distance between the distal border of the direct ACL attachment and the adjacent margin of the articular cartilage (B–G)
5. Horizontal distance between the posterior extent of the direct ACL attachment and the posterior articular surface (D–G)
6. Horizontal distance between the anterior extent portion of the direct ACL attachment and the anterior articular surface (C–H)
7. Vertical distance between the distal border of the direct ACL attachment and the inferior articular surface (B–I).

Afterwards, the ink was again completely removed, and a reference marker was used as the specimens were photographed using an 18.7-megapixel high-resolution digital camera (model EOS 1300D, Nikon Corp., Tokyo, Japan) positioned on a professional tripod (Canon Inc., Tokyo, Japan). The photograph consisted of an accurate view of the medial wall of the lateral femoral condyle perpendicular to the femoral insertion with the knees placed at 0° and 90° of knee flexion. The camera lens was placed 25 cm parallel to the articular surface, and the ACL femoral footprint was at the center of the image. In this way, a high-resolution image of each sample was recorded in case of deterioration and to be used to resolve differences between observers in the post-consensus meeting. The same images were used to calculate the area of the ACL femoral footprint and to repeat the same morphometric analyses carried out manually.

All dissections and photographs were evaluated by three independent observers. There were three knee surgeons (RMA, TACE and JCM) blind to the age and gender of the specimens. It was carried out just after the dissection and in a second revision after 2 weeks. The specimens were again evaluated in a different order from the first evaluation. Moreover, the 2nd round of evaluations was blinded to the results of the first evaluation. During the interval, the specimens were stored in a cold room for preservation. After the evaluations were concluded, the differences were reviewed in a consensus meeting using a nominal group technique without a hierarchy. The shape of the femoral footprint was described as ribbon-like (or flattened), oval/elliptical, round/circular, semicircular, small-semicircular or undetermined in accordance with the classifications previously proposed [3, 18, 19, 26, 34, 35, 37].

The images were downloaded to a personal computer. The Image J software version 1.49 (National Institute of Health, Bethesda, USA, http://rsbweb.nih.gov/ij/) was used to perform the previously described measurements and compare them with those made at the time of dissection. Moreover, the software was used to estimate the footprint area after adjusting the computer images to the actual knee size**.** The whole ACL area and the whole direct and indirect insertion area were always calculated. In cases where a precise definition of the two bundles was possible, the whole area and the direct insertion area as well as the fan-like extension fibers area of the AM and PL bundles were also calculated [16]. The accuracy of the area measurement was less than 0.1 mm and 0.1 mm^2^.

### Statistical analysis

Statistical analyses were performed using SPSS software package (version 19.0 for Windows XP). Intra and inter-rate reliability was calculated using the Cohen’s kappa and Fleiss Kappa coefficients, respectively. The Mann–Whitney *U* test was used to determine the significance of the differences between the ages of the different groups. The Chi-square test was used to compare genders within the same age group and to compare age groups between specimens of the same gender. Because we performed multiple comparisons, we used the Bonferroni correction method to calculate the appropriate alpha value for which associations can be considered statistically significant. The following formula was used to calculate the Bonferroni-corrected alpha: pre-specified alpha/number of comparisons. A *p* value of less than 0.05 was considered significant and all tests were two-tailed. An a priori sample size estimation was not done as the decision was taken to use all the samples that were available in our laboratory, regardless of the result. Post-hoc power analyses were performed based on the main outcome of finding differences between different age groups. The asymptotic relative efficiency method was performed for the Mann–Whitney *U* tests. The results are presented as median observed power and median sample required for achieving 80% power on an alfa level of 0.05 based on observed differences.

## Results

In the final analysis, 81 knees were included (45 male and 36 female). The distribution of the sample by gender and age is shown in Table 1. From the original sample of 88 knees 7 were eliminated. Advanced osteoarthritis of the tibio-femoral or patello-femoral joint was present in 3 of them, 2 had scars that were probably the result of previous knee surgery, and 2 had a complete ACL tear. The mean age of the specimens was 87 years ± 8.3 for the group of males over 50 yearsold, 28 years ± 6.2 for the group of males under 50 years of age, 82 years ± 9.2 for the females over 50 years old and 26 years ± 7.4 for the females under 50. Significant differences in the ages of the specimens from different genders were not observed. The mean weight and height of the cadaveric specimens were 79.5 kg ± 13.6 and 1.66 ± 0.08, respectively. The results of interobserver analysis were concordant (Kappa = 0.93) and the results of the intraobserver analysis were always the same (Kappa = 1). No difference was detected between the morphometric values obtained from the cadavers and from the images.

**Table 1:** Distribution of the sample by gender and age of the knees included in the study

Category	Semicircular	Ribbon-like
Male > 50 (*n* = 25)	1 (4%)	24 (96%)
Male < 50 (*n* = 20)	18 (90%)	2 (10%)
Female > 50 (*n* = 20)	1 (5%)	19 (95%)
Female < 50 (*n* = 16)	15 (93.75%)	1 (6.25%)

### ACL femoral footprint shape

**Fig. 3:**
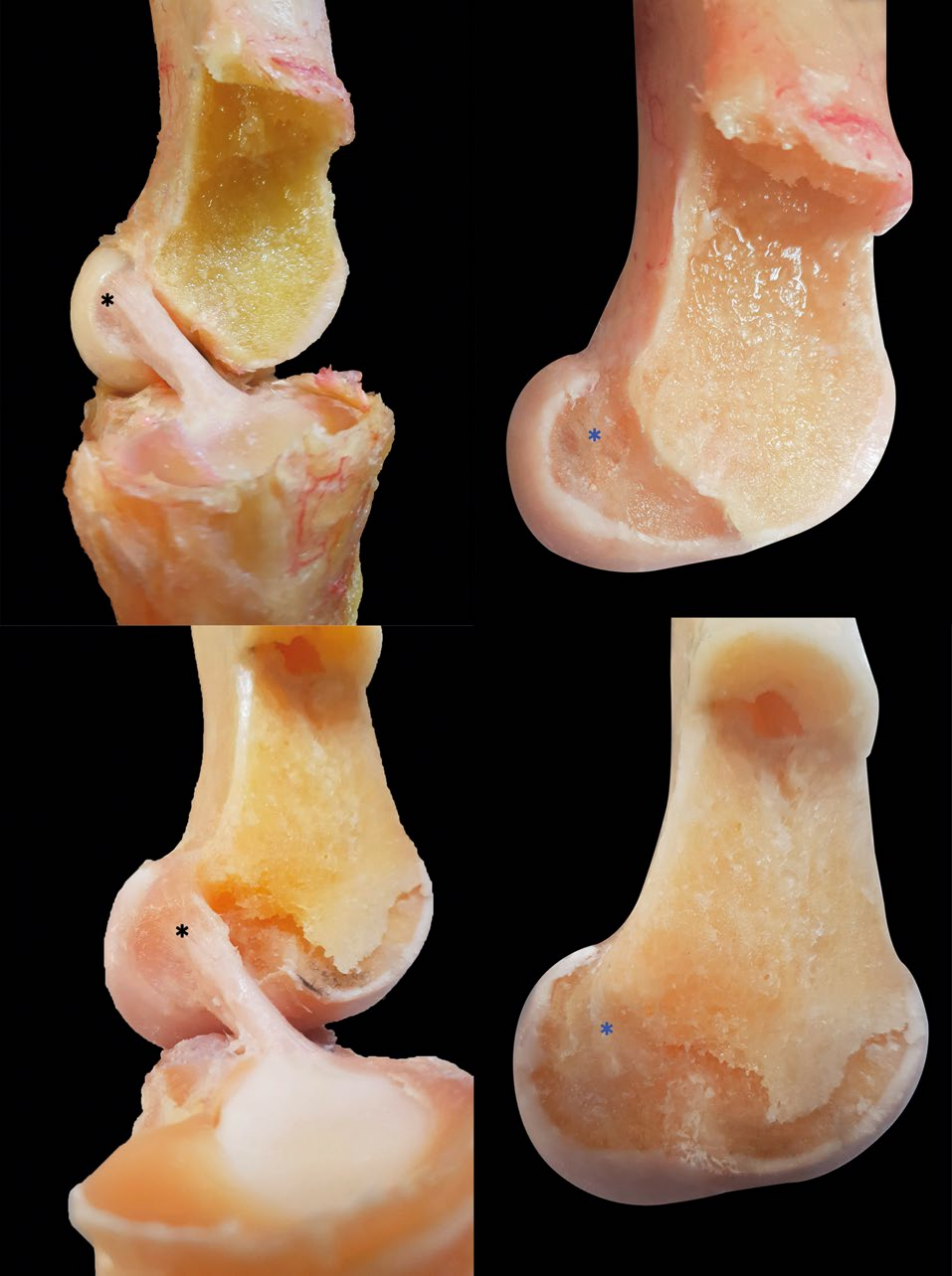
Representative images of the different ACL morphologies observed in this study before and after cutting the ACL near its femoral insertion. **A** Semicircular morphology. **B** Oval/elliptical morphology. **C** Semicircular morphology without the ligament. **D** Oval/elliptical morphology without the ligament. The asterisks (*) indicate the femoral insertion sites of the ACL in the specimens before and after the ligament is cut

The knees from the cadavers of males younger than 50 years of age presented a “semicircular” morphology in 90% (*n* = 18/20) of the cases (Fig. 3A, B). In the group of males over 50 years old, a ribbon-like appearance was founded in 96% (*n* = 24/25) of the cases (Fig. 3C, D). In the group of women less than 50 years old, the semicircular morphology was observed in 93.7% (*n* = 15/16) of the cases. In women over 50 years, the ribbon-like morphology was found in 95% (*n* = 19/20) of the cases (Table 1). No other types of morphologies previously described in the literature were found.

The chi-square test was applied to compare the results of the prevalence rates of the different observed morphologies. When independently comparing the group of men < 50 years vs males > 50 years, females < 50 years vs females > 50 years and the totality of the knees < 50 years all the knees vs > 50 years (regardless of gender), the value of *p* was < 0.001. However, no differences were observed between males and females of the same age group (Table 2).

**Table 2:** Values of *p* obtained by comparing the different study groups relative to age and gender

Comparison	*p *value
males < 50 years vs. males > 50 years	< 0.001
females < 50 years vs. females > 50 years	< 0.001
total knees < 50 years vs. total knees > 50 years	< 0.001
males < 50 years vs. females < 50 years	n.s.
males > 50 years vs. females > 50 years	n.s.
Total male knees vs. total female knees	n.s.

### Macroscopic anatomy

In young cadavers of both genders, the most prevalent morphology was semicircular, which had a straight anterior border that contacts the lateral intercondylar ridge and its posterior side convex that almost contacts the posterior articular cartilage. The indirect fibers in this group were scarce and they occupied a small area in the portion closest to the posterior articular cartilage. It was possible to identify the two ACL bundles separately in 100% of the cases. In all knees, the direct insertion site and the fanlike extensions were successfully divided. In the cadavers of people of advanced age, the direct femoral insertion moves away from the posterior edge of the articular cartilage and is kept in bounded anteriorly by the lateral intercondylar ridge, having a continuity with the posterior cortex of the femur. The insertion morphology is thinner and has the shape of a flattened ellipse, simulating a ribbon. It is smaller compared to its younger counterpart and the indirect insertion fibers are more abundant while occupying a larger area and almost come into contact with the posterior edge of the articular cartilage. In some cases (8/45), it was not possible to identify the two bundles of the ligament, individually.

### Morphometries and areas

The average measurements of the morphometries and areas of the study groups are shown in Tables 3 and 4. Relative to gender, there were no significant differences for substantially few variables. One exception was the entire area of the ACL (127 mm^2^ ± 23 in Women vs. 145 mm^2^ ± 21 in men, *p* = 0.01 and 111 mm^2^ ± 18 vs 123 mm^2^ ± 19, *p* = 0.03 for < 50 and > 50 years old, respectively). However, a pattern of greater morphometries and areas of the ACL in men than in women was observed. Moreover, the distance from the posterior edge of the insertion to the articular cartilage was always greater in women. Nonetheless, all these results were not significant (Table 3).

**Table 3:** Comparison between the average values obtained in each gender for the same age group

Category	Age range (years)	Female (mean ± SD)	Male (mean ± SD)	Female vs. male (*p* value)
Proximodistal diameter of the direct ACL attachment area (Long axis)	< 50	16.55 mm ± 2.42	17.02 mm ± 2.24	n.s.
	> 50	13.22 mm ± 1.85	15.44 mm ± 1.95	n.s.
Anteroposterior diameter of the direct ACL attachment area (Short axis)	< 50	9.32 mm ± 1.13	11.22 mm ± 1.4	n.s.
	> 50	3.12 mm ± 0.72	4.92 mm ± 0.85	n.s.
Horizontal distance between the proximal border of the direct ACL attachment and the adjacent margin of the articular cartilage	< 50	2.2 mm ± 0.55	2.0 mm ± 0.48	n.s.
	> 50	7.3 mm ± 2.4	6.7 mm ± 1.93	n.s.
Horizontal distance between the distal border of the direct ACL attachment and the adjacent margin of the articular cartilage	< 50	2.5 mm ± 0.42	2.3 mm ± 0.66	n.s.
	> 50	8.8 mm ± 1.76	8.5 mm ± 2.12	n.s.
Horizontal distance between the posterior extent of the direct ACL attachment and the posterior articular surface	< 50	2.3 mm ± 0.61	2.1 mm ± 0.59	n.s.
	> 50	8.2 mm ± 2.02	7.9 mm ± 1.75	n.s.
Horizontal distance between the anterior extent portion of the direct ACL attachment and the anterior articular surface	< 50	13.21 mm ± 1.25	14.32 mm ± 1.66	n.s.
	> 50	14.55 mm ± 1.87	15.02 mm ± 2.04	n.s.
Vertical distance between the distal border of the direct ACL attachment and the inferior articular surface	< 50	2.02 mm ± 0.88	1.55 mm ± 0.72	n.s.
	> 50	2.97 mm ± 1.22	2.43 mm ± 1.07	n.s.
Whole femoral footprint area	< 50	127 mm^2^ ± 23	145 mm^2^ ± 21	0.012*
	> 50	111 mm^2^ ± 18	123 mm^2^ ± 19	0.03
Whole direct insertion area	< 50	104 mm^2^ ± 17	122 mm^2^ ± 20	n.s.
	> 50	57 mm^2^ ± 11	61 mm^2^ ± 15	n.s.
Whole indirect insertion area	< 50	19 mm^2^ ± 6	17 mm^2^ ± 4	n.s.
	> 50	47 mm^2^ ± 14	56 mm^2^ ± 15	n.s.
Whole AM footprint area	< 50	67 mm^2^ ± 15	78 mm^2^ ± 13	n.s.
	> 50	55 mm^2^ ± 13	62 mm^2^ ± 14	n.s.
Direct insertion area of AM bundle	< 50	52 mm^2^ ± 10	63 mm^2^ ± 11	n.s.
	> 50	26 mm^2^ ± 7	32 mm^2^ ± 8	n.s.
Indirect insertion area of AM bundle	< 50	10 mm^2^ ± 3	12 mm^2^ ± 3	n.s.
	> 50	25 mm^2^ ± 5	34 mm^2^ ± 8	n.s.
Whole PL footprint area	< 50	59 mm^2^ ± 10	65 mm^2^ ± 11	n.s.
	> 50	53 mm^2^ ± 8	58 mm^2^ ± 9	n.s.
Direct insertion area of PL bundle	< 50	48 mm^2^ ± 5	51 mm^2^ ± 6	n.s.
	> 50	24 mm^2^ ± 3	29 mm^2^ ± 5	n.s.
Indirect insertion area of PL bundle	< 50	10 mm^2^ ± 1	12 mm^2^ ± 2	n.s.
	> 50	22 mm^2^ ± 5	28 mm^2^ ± 7	n.s.

We used the Bonferroni correction method to determine the *p* value at which the comparisons would be considered statistically significant due to multiple comparisons. The Bonferroni-corrected alpha value in these comparisons is 0.0125 instead of the traditional 0.05. Significant *p* values at this threshold are marked with an asterisk

**Table 4:** Comparison between the average values obtained in each age group for the same gender

Category	Gender	< 50 years (mean ± SD)	> 50 years (mean ± SD)	< 50 years vs > 50 years (*p* value)
Proximodistal diameter of the direct ACL attachment area	Female	16.55 mm ± 2.4	13.22 mm ± 1.8	n.s.
	Male	17.0 mm ± 2.2	15.44 mm ± 1.9	n.s.
Anteroposterior diameter of the direct ACL attachment area	Female	9.32 mm ± 1.1	3.12 mm ± 0.72	< 0.0001*
	Male	11.22 mm ± 1.4	4.92 mm ± 0.85	< 0.0001*
Horizontal distance between the proximal border of the direct ACL attachment and the adjacent margin of the articular cartilage	Female	2.2 mm ± 0.55	7.3 mm ± 2.4	< 0.0001*
	Male	2.0 mm ± 0.48	6.7 mm ± 1.93	< 0.0001*
Horizontal distance between the distal border of the direct ACL attachment and the adjacent margin of the articular cartilage	Female	2.5 mm ± 0.42	8.8 mm ± 1.76	< 0.0001*
	Male	2.3 mm ± 0.66	8.5 mm ± 2.12	< 0.0001*
Horizontal distance between the posterior extent of the direct ACL attachment and the posterior articular surface	Female	2.3 mm ± 0.61	8.2 mm ± 2.02	< 0.0001*
	Male	2.1 mm ± 0.59	7.9 mm ± 1.75	< 0.0001*
Horizontal distance between the anterior extent portion of the direct ACL attachment and the anterior articular surface	Female	13.21 mm ± 1.25	14.55 mm ± 1.87	n.s.
	Male	14.32 mm ± 1.66	15.02 mm ± 2.04	n.s.
Vertical distance between the distal border of the direct ACL attachment and the inferior articular surface	Female	2.02 mm ± 0.88	2.97 mm ± 1.22	n.s.
	Male	1.55 mm ± 0.72	2.43 mm ± 1.07	n.s.
Whole femoral footprint area	Female	127 mm^2^ ± 23	111 mm^2^ ± 18	0.02
	Male	145 mm^2^ ± 21	123 mm^2^ ± 19	0.0006*
Whole direct insertion area	Female	104 mm^2^ ± 17	57 mm^2^ ± 11	< 0.0001*
	Male	122 mm^2^ ± 20	61 mm^2^ ± 15	< 0.0001*
Whole indirect insertion area	Female	19 mm^2^ ± 6	47 mm^2^ ± 14	< 0.0001*
	Male	17 mm^2^ ± 4	56 mm^2^ ± 15	< 0.0001*
Whole AM footprint area	Female	67 mm^2^ ± 15	55 mm^2^ ± 13	n.s.
	Male	78 mm^2^ ± 13	62 mm^2^ ± 14	n.s.
Direct insertion area of AM bundle	Female	52 mm^2^ ± 10	26 mm^2^ ± 7	< 0.0001*
	Male	63 mm^2^ ± 11	32 mm^2^ ± 8	< 0.0001*
Indirect insertion area of AM bundle	Female	10 mm^2^ ± 3	25 mm^2^ ± 5	< 0.0001*
	Male	12 mm^2^ ± 3	34 mm^2^ ± 8	< 0.0001*
Whole PL footprint area	Female	59 mm^2^ ± 10	53 mm^2^ ± 8	n.s.
	Male	65 mm^2^ ± 11	58 mm^2^ ± 9	n.s.
Direct insertion area of PL bundle	Female	48 mm^2^ ± 5	24 mm^2^ ± 3	< 0.0001*
	Male	51 mm^2^ ± 6	29 mm^2^ ± 5	< 0.0001*
Indirect insertion area of PL bundle	Female	10 mm^2^ ± 1	22 mm^2^ ± 5	< 0.0001*
	Male	12 mm^2^ ± 2	28 mm^2^ ± 7	< 0.0001*

We used the Bonferroni correction method to determine the *p* value at which the comparisons would be considered statistically significant due to multiple comparisons. The Bonferroni-corrected alpha value in these comparisons is 0.0125 instead of the traditional 0.05. Significant *p* values at this threshold are marked with an asterisk

Most of the distances between the articular cartilage and the direct femoral insertion of the ACL (the distances up to the posterior articular cartilage) were significantly greater in the older group. At the same time, it was noted that the anteroposterior diameter of the direct insertion of the ACL significantly decreased in both genders with the passage of time. The ACL as well as all the areas (direct and indirect) of the AM and PL fascicles do not present significant variations with respect to age. However, the proportion between the direct and indirect areas between the younger and older knees does show significant variations over time. The whole area of the ACL does not show significant variations but the proportion between their direct and indirect insertion areas changed with aging, the direct areas being significantly higher in the under 50 groups and the indirect insertion areas significantly greater in the over 50 groups (Table 4).

Relative to the result from the post-hoc power analyses, the median power achieved across comparisons between age groups was 0.99 (0.81–0.99), with median sample size requirements of 4 (3–10) cadavers per group. This confirms the validity of the acquired sample for this study.

## Discussion

The main finding of the present study was that the femoral insertion of the ACL presents variations in its morphology over time. It goes from being large and semicircular in shape in young subjects to a smaller and flattened ribbon-like shape in older subjects, which confirms the initial hypothesis. In the same way, significant differences were detected in most of the morphometric parameters and areas analyzed between the different age groups of the same gender. Additionally, there were no differences between genders in the same age group.

One type of tissue at the femoral attachment was described as dense mid substance fibers that are attached to a narrow oval area on the lateral condyle (direct insertion). Another was a thin, membranous, peripheral attachment of fibers that spread out towards the posterolateral condyle, which increases the area of the insertion site (a fan-like extension or indirect insertion site) [33].

The shape of the femoral footprint was described as ribbon-like (or flattened) [37], oval/elliptical, [3, 18, 19, 21, 26, 34, 35, 38] round/circular [10, 12], or semicircular [7]. In this study, a significant difference between the morphology was seen between cadavers of those younger and older than 50 years. In the older than 50, a thin structure with its greater diameter from proximal to distal in the shape of a ribbon-like was observed, similar to the one described by Smigielski [37] in a study with cadavers with a mean age of 67 years. In people younger than 50, a large semicircular structure, similar to the one described by Ferretti [7], was found. The latter study was conducted on specimens with a mean age of 75, and the shape differs from the one in the present study given that an anterior border straight and a posterior border convex have been described. Conversely, most authors have described an ovoid or elliptical insertion area [3, 18, 19, 21, 26, 34, 35, 38]. All those studies were conducted on cadavers of people with a mean age that was greater than 50 years. Thus, more studies on the cadavers of younger people need to be done to confirm the observed morphology.

Due to the decrease in the size of the direct insertion with increasing age, the mean distance from the insertion of the ACL to the posterior cartilage of the lateral femoral condyle increases from 2.3 to 8.2 mm (*p* =  < 0.0001) in women, and from 2.1 to 7.9 mm in men (*p* =  < 0.0001). On the contrary, the morphometries towards the lower and anterior cartilage do not suffer significant changes. In this study, the direct insertion of the ACL decreases at its anteroposterior axis, which causes this structure to become thinner or flattened in patients older than 50, similar to previous studies [37, 39].

On the other hand, the difference between the different age groups in the areas corresponding to the direct and indirect insertions of the ACL is great. We hypothesize that this indirect insertion occurs as a consequence of the degeneration of the fibers of direct insertion of the ACL over time. Additionally, our findings indicate that both fascicles of the ACL contribute to the decrease in size of the direct insertion area. In this regard, the AM bundle in female was 52 mm^2^ in cadavers under 50 years and 30 mm^2^ in cadavers older than 50 years old, while it was 59 mm^2^ vs 32 mm^2^ in males. The same significant effect was observed in the posterolateral fascicle. Similarly, an increase in the area of indirect insertion was observed in both fascicles in older cadavers with the AM bundle being 10 mm^2^ in females younger than 50 and 29 mm^2^ in females older over 50. The males presented 11 mm^2^ in the cadavers of those under 50 vs. 34 mm^2^ in the over 50-year-old group.

Otherwise, unlike the changes that occur between direct and indirect insertion as they age, there is no statistically significant difference between the total size of the femoral insertion of both fascicles between age groups. It was 145 mm^2^ and 123 mm^2^, respectively in males and 127 mm^2^ and 111 mm^2^ in females. Contrarily, a different proportion was found between the direct and indirect portion in both age groups. The direct insertion of the whole area goes from 84% in males under 50 years of age to 49% in those over 50 years of age, while it goes from 90 to 51% in females, respectively. The hypothesis put forward in this work is that the direct insertion suffers a degeneration in both fascicles and the indirect insertion area increases with aging.

Therefore, an area of direct femoral insertion similar to that reported in the literature was observed in the cadavers of those over 50-year-old. In the present study, it was 57 mm^2^ (SD ± 11) in females and 61 mm^2^ (SD ± 15) in males, similar to those reported by previous studies (56 mm^2^,[16] 61 mm^2^,[19] 50.8 mm^2^ [25] and 67 mm^2^ [39]). This is consistent with the fact that all previous cadaver studies where the femoral footprint was measured have presented a mean age that was greater than 50. The reported age ranges from 57 to 83 years of age. Araki et al. reported a mean age of 62.0 years [1], Colombet et al. 75 years [3], Fujimaki et al. 57.5 years [8], Gali et al. 74 years [9], Hart et al. 82.5 years [13], Iriuchisima et al. 82.5 years [16] and 83 years [17], Iwama et al. 80 years [19], Mochizuki et al. 61.3 years [25], Sasaki et al. 69.8 years [34], Siebold et al. 82 years, [36] Suruga et al. 83 years, [39 years and 78.8 years [38]], Kim et al. 70 years, [20] and Tampere et al. 81.5 years [41]. These results are evidently lower than what we observed in patients under 50 years of age, where they presented a larger direct femoral insertion area; 114 mm^2^ (SD ± 17) in females and 122 mm^2^ (SD ± 20) in males. This was a statistically significant difference compared to patients older than 50 years of age. No studies were found with a large direct insertion area similar to the results of this study.

Here, a significant increase in the indirect insertion area was observed in patients over 50 years of age, presenting 50 mm^2^ (SD ± 14) in women and 56 mm^2^ (SD ± 15) in men, similar to that observed in previous studies. Suruga et al. reported an area of 59 mm^2^ in patients with a mean age of 83 years [39], and Iriuchishima et al. reported 45 mm^2^ with a mean age of 82.5 years [16]. In addition, it was observed that indirect fibers have a smaller insertional area in the cadavers of those under 50 years of age when compared to the over-50 cadavers, showing that indirect fibers increase with age. This effect may be due to a degeneration of the direct fibers into fan-like fibers as age increases.

There are previous studies that showed degenerative histopathologic changes in the ACL bundles that occur with aging in osteoarthritic knees, presenting a weaker and thinner ligament in those patients [4, 14, 28] that may explain the degeneration of the mid substance insertions on the femoral condyle. A recent study demonstrated that both fascicles of the ACL presented histologic changes. The AM bundle showed degenerative changes in 53% of the specimens and the PL bundle showed degeneration in 78% of the knees. The PL fascicle also presented more severe degenerative changes [41], suggesting that intercondylar notch stenosis and intercondylar notch osteophytes led to ACL destruction [15, 22].

For this reason, describing the correct size and morphology of the ACL footprint is critical to reproducing the native anatomy as closely as possible. It is important to consider this change in the configuration of the ACL morphology as age advances for carrying out femoral tunnels in ACL reconstruction. Subsequent studies with cadavers divided into decadal subgroups, for example, are needed to confirm our results [5, 30].

These findings should be considered to avoid employing reconstruction techniques in which femoral tunnels with oval or rectangular shapes are used in patients under 50 years of age. They do not fit with the morphology of the femoral insertion of the ACL in this age group.

Despite the rigorous methodology followed, certain limitations of the study must be acknowledged. One is that the use of cadavers belonging to a single population does not allow us to generalize our results for all populations. Moreover, the lack of a histological analysis to precisely delimit the limit between the direct and indirect fibers of the ACL is another limitation. There is also the use of embalmed cadavers, which has not previously been associated with alterations in the morphological patterns of bones and ligaments. However, the use of fresh-frozen specimens would be ideal. Finally, another limitation is that we did not perform a sample size estimation a priori because we worked with all the samples available in our laboratory. Nonetheless, post-hoc power analyses indicated that our sample was adequate. Moreover, we divided our initial sample size into four groups to perform hypotheses testing, which further reduced our power. However, to mitigate this type of error, we used a Bonferroni-corrected alpha value to denote statistical significance when we performed multiple comparisons. A decision was also taken to use non-parametric statistical tests for a more conservative statistical approach.

## Conclusions

The femoral insertion of the ACL presents variations in its morphology, area and morphometric characteristics over time. It goes from a large semicircular shape that almost contacts the posterior articular cartilage in the younger group to a smaller, flattened and ribbon-like shape that moves away from the edge of the articular cartilage and is kept in bound anteriorly by the lateral intercondylar ridge in the older age group.
